# Suppressive CD8+ T‐Cells Are Key Cellular Mediators of Extracorporeal Photopheresis

**DOI:** 10.1002/jca.70094

**Published:** 2026-02-07

**Authors:** Kai J. Rogers, Kathryn L. Eschbacher, Zeb Zacharias, Kshitija Kale, Michael P. Crawford, Charles Michael Knudson, Alexander W. Boyden, Nitin J. Karandikar

**Affiliations:** ^1^ Department of Pathology University of Iowa Health Care Iowa City Iowa USA; ^2^ Translational Immunology Core University of Iowa Health Care Iowa City Iowa USA; ^3^ Holden Comprehensive Cancer Center University of Iowa Health Care Iowa City Iowa USA; ^4^ Iowa City Veterans Affairs Medical Center Iowa City Iowa USA

**Keywords:** apheresis, CD8^+^ T‐cell, experimental autoimmune encephalomyelitis, extracorporeal photopheresis, multiple sclerosis

## Abstract

Extracorporeal photopheresis (ECP) is a widely utilized immunomodulatory procedure with an incompletely defined mechanism. In graft‐versus‐host disease (GvHD) and transplant rejection, ECP is thought to induce immune tolerance by increasing regulatory CD4+ T‐cells, whereas in cutaneous T cell lymphoa it may enhance dendritic cell–mediated antigen presentation and cytotoxic T cell activity. We investigated the role of CD8+ T cells in ECP using a murine model of multiple sclerosis (MS). ECP protected mice from disease, mitigated CNS pathology, and was dependent on CD8+ T cells. Translation to patients revealed increased numbers of suppressive CD8+ T‐cells. Functional assays identified enhanced suppressive capacity of CD8+ T‐cells in ECP patients and longitudinal studies found this occurred within 1 month of starting ECP. Using both a murine model and clinical samples, our findings reveal a mechanistic role for suppressive CD8+ T‐cells in mediating the effects of ECP, potentially providing a unifying mechanism for ECP's apparently dichotomous effects.

## 1 Introduction

The immunomodulatory procedure extracorporeal photopheresis (ECP) has been used clinically since the 1980s. However, the mechanism by which it exerts its effect remains poorly understood. Originally indicated for the treatment of cutaneous T cell lymphoma (CTCL), specifically mycoses fungoides and its circulating form Sezary syndrome, the use of ECP in routine practice has since expanded to include the treatment of graft‐versus‐host disease (GvHD), systemic sclerosis, and solid organ transplant rejection. More experimentally, ECP has been used for the treatment of autoimmune disorders such as Crohn's disease, systemic lupus erythematosus, and Type 1 diabetes. Mechanically, ECP involves the collection of leukocytes (the “buffy coat”) from a portion of the patient's blood. A photosensitizing agent is then added, and the cells are exposed to UV light before ultimately being returned to the patient, a step that was found to be essential for clinical benefit [1, 2]. While much work has been done to elucidate the mechanism by which ECP works [3, 4, 5, 6], no consensus has been reached. The leading hypothesis is that ECP induces monocyte differentiation to dendritic cells that then present autoantigen, presumably in conjunction with immunomodulatory cytokine production, leading to the formation of regulatory T cells (Tregs) that induce immune tolerance [5].

While most of the scientific literature focuses on CD4+ Tregs, a growing body of evidence has found that CD8+ T cells can also adopt a regulatory phenotype. Such cells have been implicated in a variety of autoimmune disorders [7, 8], and our laboratory has explored these cells in the context of multiple sclerosis (MS) [9, 10, 11, 12, 13] and its mouse model experimental autoimmune encephalomyelitis (EAE) [14, 15, 16, 17, 18]. Interestingly, a unique suppressive CD8+ T cell population has recently been implicated in a murine model of contact hypersensitivity treated with ECP [19].

To directly explore the role of CD8+ T cells in ECP we adapted a murine model of ECP [20] to EAE, a well‐established laboratory model that simulates many of the inflammatory aspects of MS [21, 22] which our laboratory has utilized extensively [16, 23, 24, 25]. Using this model system, we found that ECP reduced the severity of EAE and that it did so when administered before or after symptom onset. ECP also reduced neuroinflammation and demyelination in the brain and spinal cord of treated animals. This protective effect was highly antigen dependent and found to be reliant on CD8^+^ T cells. We then correlated these findings to human patients and found that suppressive CD8^+^ T cells are increased in ECP patients relative to healthy donors. Additionally, we found that ECP treatment rapidly enhances the suppressive capacity of CD8+ T cells. Taken together, these studies provide mechanistic insight into ECP, while providing a basis for potentially expanding its reach as a targeted immunotherapy for autoimmune neuroinflammatory disorders.

## 2 Methods

### 2.1 Informed Consent and Ethics Statement

All human experiments were performed on peripheral blood mononuclear cells (PBMCs) obtained from patients undergoing ECP or healthy platelet donors who were consented at our institution's Blood Center, as approved by the institutional IRB (Biorepository for assessment of changes in immunity in patients treated with ECP, IRB # 202206379).

### 2.2 Mice

6–8‐week‐old female SJL/J (Strain #000686), C57BL/6J (Strain #000664), and CD8^−/−^ C57BL/6 (Strain #002665) mice were purchased from The Jackson Laboratory (Bar Harbor, ME). All experimental procedures were approved by and performed in accordance with the University of Iowa's Animal Care and Use Committee (IACUC).

### 2.3 EAE Induction

Classical EAE was induced as previously described by our laboratory [24]. Briefly, mice were immunized with 50 μg of PLP_178–191_ (NTWTTCQSIAFPSK), PLP_139–151_ (HSLGKWLGHPDKF), or MOG_35–55_ (MEVGWYRSPFSRVVHLYRNGK) peptides (purchased from GenScript, Piscataway, NJ) emulsified in a 1:1 volume with complete Freund's adjuvant (CFA) supplemented with 4 mg/mL *Mycobacterium tuberculosis* (Becton Dickinson, Franklin Lakes, NJ). The resulting emulsion was injected subcutaneously in two 50 μL aliquots in the bilateral flanks. Mice subsequently received 250 ng of pertussis toxin (ptx) in glycerol (List Labs, Campbell, CA) administered on day 0 and again on day 2 after immunization to facilitate cell trafficking across the blood brain barrier and induce EAE. Mice receiving PLP_139–151_ did not receive ptx as this is not required to induce EAE in this model. Clinical scores were assessed daily in a blinded manner, and animals were scored using previously defined criteria [26]:

### 2.4 Clinical Score

0 = normal mouse, 1 = limp tail, 2 = mild hind limb weakness, 3 = moderate hind limb weakness/partial paralysis, 4 = bilateral complete hind limb paralysis, 5 = moribund.

### 2.5 ECP

Our ECP protocol is based on a similar approach used by others [27]. Donor mice were immunized (no ptx given) with the indicated autoantigen or OVA_323‐339_ (ISQAVHAAHAEINEAGR, GenScript, Piscataway, NJ) antigen 12 days prior to cell harvest. Mice were euthanized, spleens and bilateral inguinal lymph nodes were harvested, and a single cell suspension was generated by passing cells through a 100 μm mesh strainer. RBCs were lysed and cells were resuspended in a complete growth medium. Cells were incubated at 37C with 200 ng/mL methoxsalen (MedChemExpress) for 1 h. Cells were then exposed to a total 2.5 J/cm^2^ UVA light (315–400 nm) using the BS‐02 irradiation chamber with UV‐MAT (Opsytec Dr. Groebel, Ettlingen, Germany). Cells were allowed to rest at 37C for 1 h prior to transfer to recipient animals. All mice undergoing ECP treatment received a single dose of 10 × 10e6 cells given via retro‐orbital injection. In experiments evaluating cell subsets that mediate ECP, cells were split into CD3+ and CD3− populations using the EasySep Mouse T Cell Isolation Kit (Stemcell Technologies, 19 851) prior to transfer.

### 2.6 Histologic Assessment of Disease

Female C57BL/6J mice were induced for EAE using the MOG_35‐55_ antigen. On day 15 after induction, mice were provided to the University of Iowa's Comparative Pathology Laboratory (CPL) at our institution. Mice were euthanized, brain and lumbar spinal cord were harvested, and slides were cut from formalin fixed paraffin embedded tissue blocks. Slides were stained with either hematoxylin and eosin (H&E) (to assess presence of neuroinflammation) or luxol fast blue (LFB) (myelin stain). A blinded neuropathologist assessed the slides and scored them for demyelination, axonal swellings, and inflammation according to the following criteria [25]:

Demyelination: 0 = None, 1 = Rare foci, 2 = A few areas, 3 = Large confluent areas.

Axonal Swellings: 0 = no dilated axonal sheaths and/or spheroids, 1 = rare, scattered, and individualized empty dilated axonal sheaths and/or spheroids, 2 = 1–3 foci of small clusters of empty dilated axonal sheaths and/or spheroids, 3 = greater than three foci of small to large clusters of empty dilated axonal sheaths and/or spheroids.

Inflammation: 0 = no inflammation, 1 = 1–4 small foci of scattered inflammatory cells, 2 = greater than five small foci of inflammatory cells or 1–3 foci of moderate numbers of inflammatory cells including perivascular organization, 3 = 4 or more foci of moderate numbers of inflammatory cells, extensive perivascular cuffing, or one or more collections of abundant number of inflammatory cells.

### 2.7 Adoptive Transfer Experiments

Adoptive transfers were performed as previously described [16]. Briefly, donor mice were immunized with the indicated antigen in CFA. At 12 days post‐immunization, splenocytes and inguinal lymph nodes were harvested, and cells were restimulated in vitro with 20 μg/mL cognate antigen and 10 pg/mL rIL‐2 for 72 h in complete RPMI. CD8+ T cells were sorted using the EasySep Mouse CD8+ T Cell Isolation Kit (Stemcell Technologies, 19 853) and 10 × 10^6^ cells were adoptively transferred by intravenous injection into recipient mice at times indicated.

### 2.8 PBMC Isolation and Cell Sorting in Human Subjects

PBMCs were isolated from ~28 mL of peripheral blood collected in EDTA anticoagulated (purple top) tubes by nursing staff in the DeGowin Blood Center. Peripheral blood samples were processed within 4 h of collection. Cells were separated from buffy coats using Ficoll Hypaque (GE Healthcare Biosciences, Pittsburg, PA) density gradient. Cells that were not used immediately were stored in DMSO‐containing media in liquid nitrogen for later use. Frozen cells (used for experiments comparing specified timepoints) were subsequently thawed in RPMI 1640 (Corning, 10–040‐CV) with DNase at 10KU/ml (Sigma D4513–1vl) and confirmed to be at least 90% viable. Fresh or thawed cells were subsequently sorted according to the following scheme. First, bulk CD8+ T cells were isolated by positive selection using the CD8+ T Cell Isolation Kit (Miltenyi Biotec, 130‐096‐495). CD4+ CD25− cells were subsequently isolated from the CD8‐depleted cell population by negative selection using the CD4+ T Cell Isolation Kit (Miltenyi Biotec, 130‐096‐533) and CD25 Microbeads (Miltenyi Biotec, 130‐092‐983). Once sorted, cells were immediately used for suppression assays as described below.

### 2.9 Suppression Assays

CD4+ T cells from ECP patients or healthy controls were used for flow cytometric suppression assays, as described previously [10, 13]. Briefly, responder CD4+ CD25− T cells were stained with CFSE, followed by culture in 96‐well plates with 1 μg/mL anti‐CD3 (eBioscience, 16‐0037‐85) and anti‐CD28 (eBioscience, 16‐0288‐38), previously fixed on the plate for 1 h at 37C and then aspirated immediately prior to the addition of cells. CD4+ T cells were incubated in the presence or absence of bulk CD8+ T cells (autologous or allogeneic as indicated) in RPMI 1640 with 5% human serum, 1% Pen/Strep, and 1% L‐glutamine. On day 6–7 of culture, cells were stained for anti‐CD4 PE‐Cy7 (BD, 557852), anti‐CD3 AlexaFlour700 (BD, 557943), anti‐CD8 Pacific Blue (Biolegend, 344 718) and assessed for CD4 proliferating fraction (identified as CFSE dilute cells) by flow cytometry (Cytek Aurora). Percent proliferation and percent suppression were calculated as described previously [10].

### 2.10 Figures and Statistical Analysis

Figures were generated with GraphPad Prism 7.0 (La Jolla, CA). Statistics were performed within the software. For all comparisons, Student's *T*‐test was used. *p* values < 0.05 were considered significant and indicated as follows: * = *p* < 0.05, ** = *p* < 0.01, and *** = *p* < 0.001.

## 3 Results

### 3.1 ECP Reduces the Severity of EAE in Mice

**FIGURE 1:**
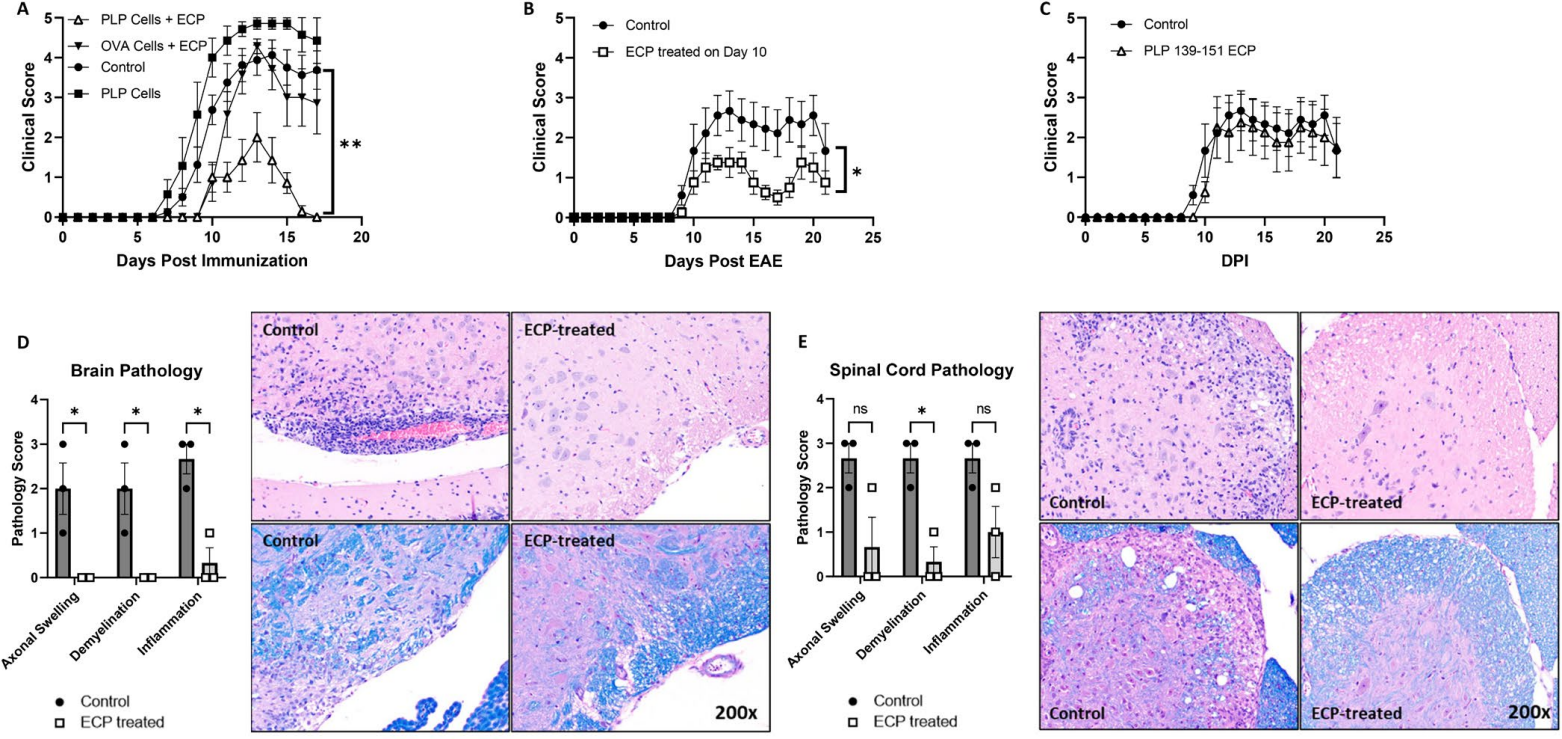
ECP reduces the severity of EAE. For all experiments 6–8 week old female mice of the indicated strain were used. EAE was induced with the indicated peptide on Day 0 and mice were monitored daily for signs of disease. (A) ECP was performed using spleen + LN cells obtained from OVA or PLP_178‐191_ immunized mice on Day 12 post immunization. 10 million cells were transferred to recipient mice on Day 0. Control mice received no cells, “PLP cells” mice received untreated cells from immunized mice (no ECP). *N* = 7 mice/group. (B) ECP was performed using spleen + LN cells obtained from PLP_178‐191_ immunized mice on Day 12 post immunization. 10 million cells were transferred to recipient mice on Day 10. Control mice received no cells. *N* = 8 mice/group. (C) ECP was performed using spleen + LN cells obtained from PLP_139‐151_ immunized mice on Day 12 post immunization. 10 million cells were transferred to recipient mice on Day 0 and EAE was induced with PLP_178‐191_. Control mice received no cells. *N* = 8 mice/group. (D, E) C57B/6 mice were induced for EAE with MOG_35‐55_. A subset of mice received 10 million ECP treated cells from mice previously immunized with MOG_35‐55_. On day 15 after EAE induction, 3 mice from each group were sacrificed, and the brain and spinal cords were harvested. Sections of paraffin embedded tissues were prepared and stained with H&E or Luxol Fast Blue. Pathology was assessed in the brain (D) and spinal cord (E) according to the scoring criteria provided in the materials and methods. Statistics were performed using Student's *t*‐test with * indicating *p* < 0.05 and ** indicating *p* < 0.01.

To investigate the mechanism of ECP, we first established a robust experimental model. For this, we turned to EAE, a murine model that replicates the neuroinflammatory aspects of human MS [28] and one that our laboratory has utilized extensively. We first used the model in female SJL/J mice, where EAE is induced by immunization with a peptide fragment of proteolipid protein (PLP_178‐191_, P178), a component of the myelin sheath. First, ECP donor mice were immunized with P178 or a control peptide (chicken ovalbumin, OVA). After 12 days, the spleen and inguinal lymph nodes were harvested from immunized mice, and a portion of the total cells were treated with ECP. Following collection and treatment of cells, a second cohort of mice were induced for EAE with P178. At the time of induction, mice received no cells (control), cells from P178 or OVA immunized mice that were treated with ECP, or untreated cells from P178 immunized mice (PLP cells). Using this model, we found that a single dose of ECP‐treated cells from P178 immunized mice ameliorated the severity of EAE when administered at the time of induction (Figure 1A). We next sought to determine if ECP could be used to treat disease after symptom onset, a condition more accurately mimicking its clinical use in humans. We induced P178 EAE in mice and monitored them until the first signs of disease (Day 10), at which point we divided them into two cohorts with an approximately equivalent distribution of sickness scores and administered ECP‐treated cells from previously immunized littermates. We found that ECP treatment was able to significantly reduce disease severity even when administered after symptom onset (Figure 1B). Finally, we evaluated whether the effect of ECP was antigen specific. We immunized SJL/J mice with PLP_139‐151_ (P139), performed ECP, and transferred cells to mice induced for P178 EAE (antigen incompatible). Using this approach, we found that ECP treatment did not impact the course of EAE when the immunizing and inducing antigens were non‐identical (Figure 1C). These results suggest that the protective effect of ECP is highly specific to the dominant antigenic response, consistent with the proposed mechanism of ECP as reported by others [5, 29, 30].

Given the finding that ECP reduced disease severity in EAE, we next asked whether there was a similar reduction in disease in the CNS. To do this, we utilized a second model of EAE that is induced by immunization with a peptide fragment of myelin oligodendrocyte glycoprotein (MOG_35‐55_), which causes robust non‐remitting disease. We treated a subset of MOG EAE induced animals with a single dose of ECP and sacrificed animals on day 15 (peak disease) for histologic analysis. Sections of the brain and lumbar spinal cord were stained with H&E as well as LFB and blindly evaluated by a neuropathologist. Pathology was scored according to the scale in the Materials and Methods with higher numbers indicating more severe disease. We found that ECP treated mice had significantly reduced pathology in the brain across all metrics and significantly less pronounced demyelination in the spinal cord relative to untreated controls (representative images shown in Figure 1D,E along with cumulative data in bar graphs).

### 3.2 The Protective Effect of ECP in EAE Is Dependent on CD8+ T Cells

Once we had confirmed that ECP could effectively treat mice induced with EAE, we next sought to investigate the necessary components of the immune response in the recipient of ECP‐treated cells. Our previous studies have indicated an important role for myelin‐specific CD8+ T‐cells in lowering disease in these models. In addition, a recent paper has also identified a population of CD8+ T cells as important for protecting mice in a contact hypersensitivity model of ECP [19]. Therefore, we decided to ask whether CD8+ T cells were important in our EAE‐ECP model.

**FIGURE 2:**
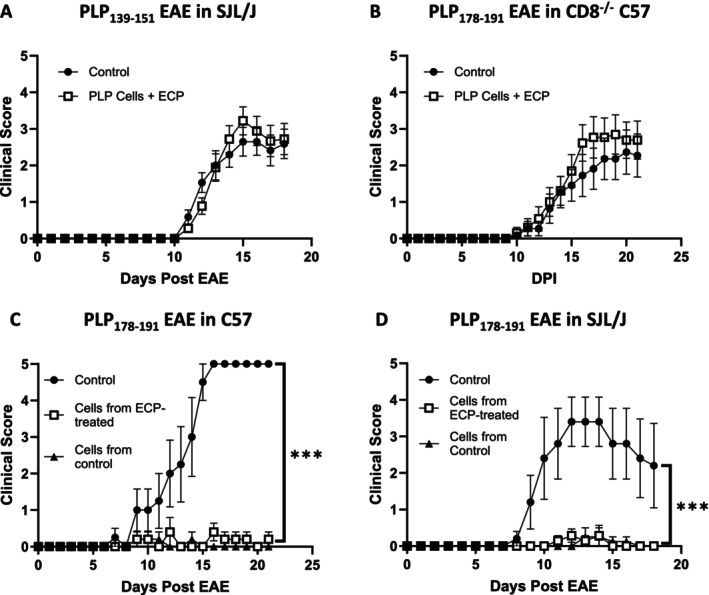
The protective effect of ECP is dependent on CD8+ T cells. For all experiments 6–8 week old female mice of the indicated strain were used. EAE was induced with the indicated peptide on Day 0 and mice were monitored daily for signs of disease. (A) ECP was performed using spleen + LN cells obtained from PLP_139‐151_ immunized mice on Day 12 post immunization. 10 million cells were transferred to recipient mice on Day 0. Control mice received no cells. *N* = 17 mice/group. (B) ECP was performed using spleen + LN cells obtained from PLP_178‐191_ immunized mice on Day 12 post immunization. 10 million cells were transferred to recipient CD8^−/−^ mice on Day 0. Control mice received no cells. *N* = 11 mice/group. (C, D) ECP was performed using spleen + LN cells obtained from PLP_178‐191_ immunized mice on Day 12 post immunization. 10 million cells were transferred to recipient mice on Day 0. Control mice received no cells. On Day 14 of EAE, mice were euthanized, spleens and LNs were harvested, and cells were incubated with IL‐2 and PLP_178‐191_ for 72 h. CD8+ T‐cells were isolated, and five million cells were transferred to recipients at the time of EAE induction with PLP_178‐191_. Control mice received no cells (control), the remainder of the mice received CD8+ T cells obtained from ECP treated (cells from ECP‐treated) or untreated (cells from control) mice. *N* = 6 mice/group. Statistics were performed using Student's *t*‐test with *** indicating *p* < 0.001.

To explore this, we first used a model of EAE induced by a third peptide, P139 in SJL/J mice. Our previous studies have shown that P139‐induced CD8+ T‐cells do not have suppressive function in P139‐induced disease, in contrast to the P178 model [17, 24]. Using this CD8‐suppressor resistant EAE model, we found that ECP was unable to mitigate disease (Figure 2A). This provided corroborative suggestion that ECP‐driven disease suppression may also be mediated through CD8+ T cells.

Given this finding, we directly investigated the role of CD8+ T cells in mediating the effects of ECP, by inducing MOG EAE in CD8‐knockout mice and attempting to protect them using ECP‐treated cells from wild‐type (WT) mice, which have CD8+ T cells. Our results showed that ECP was again unable to protect these animals (Figure 2B). Given these findings, we next sought to evaluate the protective capacity of CD8+ T cells from mice treated with ECP. We performed adoptive transfers using CD8+ T cells obtained from mice that received ECP‐treated cells or controls (no cells) and found these cells effectively prevented disease (relative to mice that did not receive CD8+ cells from either source) when transferred to recipient mice on two different genetic backgrounds (Figure 2C,D).

### 3.3 Suppressive CD8+ T Cells Are Increased in Patients Undergoing ECP Treatment

**FIGURE 3:**
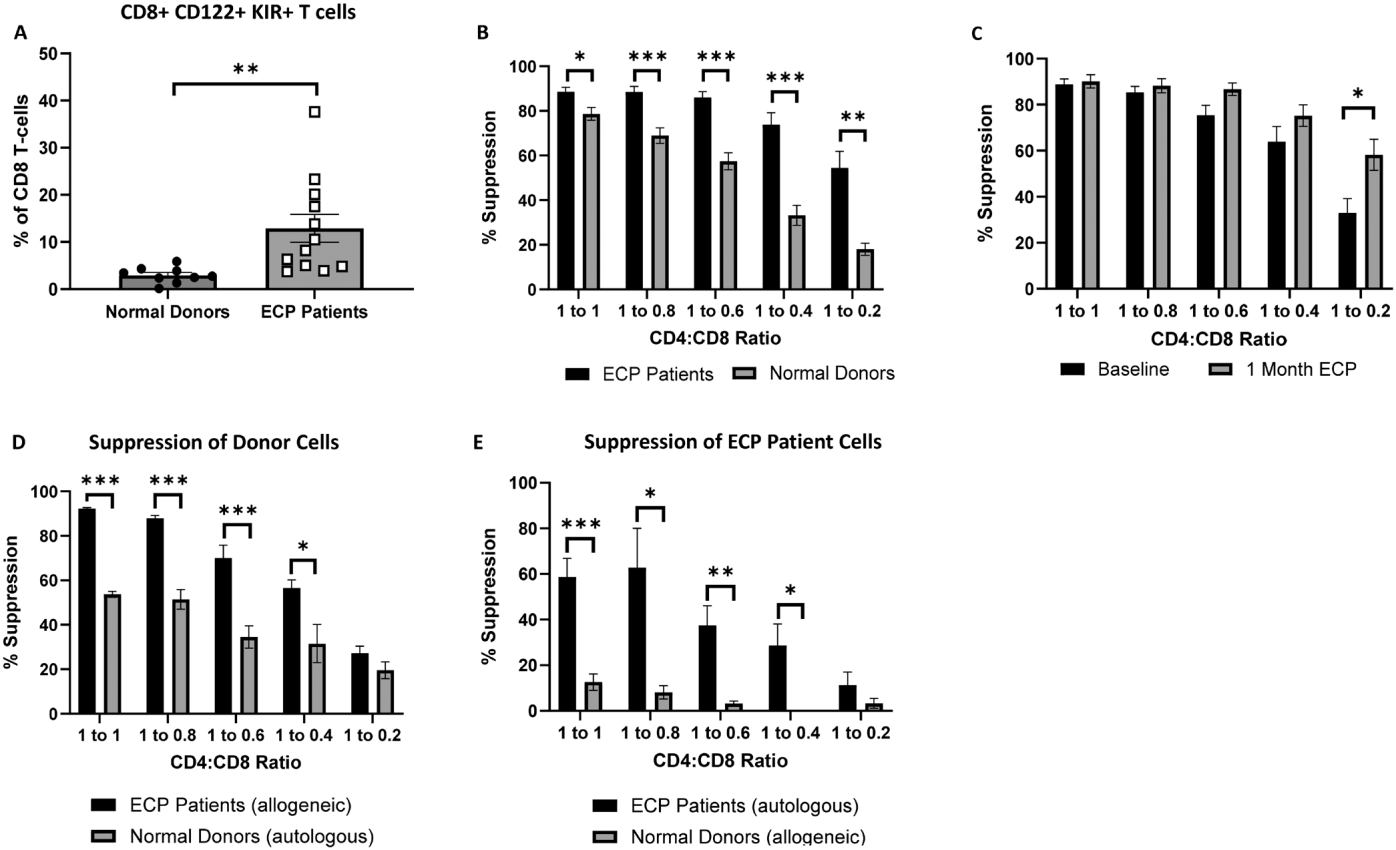
Suppressive CD8+ T cells are increased in patients undergoing ECP. For all experiments PBMCs were isolated from whole blood obtained from healthy donors or patients undergoing ECP. (A) Flow cytometry was performed, and CD8+ suppressor cells were identified as indicated. (B–E) CD4+ CD25− cells were isolated from PBMCs, stained with CFSE, and incubated with CD8+ cells at the indicated ratios for 5–7 days. Flow cytometry was performed to identify CFSE dilute CD4+ cells (divided) and the % suppression was calculated by comparing the divided populations to CD4+ cells that were incubated in the absence of CD8+ cells (more details in the materials and methods). (B) CD8‐suppression assays in healthy donors versus ECP patients. (C) CD8‐suppression assays in paired samples obtained prior to the start of ECP (Baseline) or after 1 month of therapy. (D, E) Allo‐suppression assays. CD4+ CD25− cells were isolated from healthy donors or ECP patients. Each donor was paired with an ECP patient to allow for direct comparison between autologous and allogenic suppression results. (D) CD4+ CD25− cells from the healthy donors were incubated with CD8+ cells from the same healthy donor (auto) or a paired ECP patient (allo). (E) CD4+ CD25− cells from the ECP patients were incubated with CD8+ cells from a paired healthy donor (allo) or the same ECP patient (auto). Suppression of cell proliferation was evaluated as described above. Statistics were performed using Student's *t*‐test with * indicating *p* < 0.05, ** indicating *p* < 0.01, and *** indicating *p* < 0.001.

We next wanted to see whether our observations from this mouse model would corroborate with data from human patients. To do this, we collected PBMCs from 12 patients who were undergoing routine ECP treatment (Table 1), and 9 random platelet donors who had passed all donor screening criteria (healthy controls). We performed flow cytometry to evaluate T‐cell populations, with a focus on suppressive T‐cells. We found that CD8^+^ Tregs, identified by expression of CD122 and KIR [8], were significantly increased in the cohort undergoing ECP treatment (Figure 3A). While these were interesting results, we recognized the need for functional assays to assess qualitative differences between these two populations. For this, we utilized “CD8 suppression assays” that have been widely utilized by our laboratory [10, 13]. These assays compare the proliferation of CFSE‐labeled CD4+ CD25− T cells in response to a non‐specific stimulation (anti‐CD3/CD28) in the presence or absence of bulk CD8+ T cells and across a wide range of cell ratios (Figure S1). Using this assay, we found that CD8+ T cells from ECP patients were more functionally suppressive compared to cells from healthy donors, particularly at high CD4:CD8 ratios (Figure 3B). While these were suggestive of the ability of ECP to induce CD8 suppressors, this comparison did not account for differences in these populations due to the presence of disease and/or other treatment modalities. To home in on the effect of ECP, we compared the suppressive capacity of paired CD8+ T cell populations from the same patients immediately prior to starting ECP (baseline) and after 1 month of therapy. We found that suppressive capacity in both groups was high, but significantly more pronounced at high CD4:CD8 ratios after 1 month of therapy (Figure 3C).

**TABLE 1:** ECP patient demographics.

Patient number	First ECP	Sample collection date	Age at consent	Sex	Diagnosis	Last ECP	Procedure # at sample collection	Disease status	Immunosuppressants at initiation of ECP
1	11/30/2022	4/6/2023	30	Male	GVHD	12/1/2023	25	Improved	Methylprednisolone; Budesonide; Tacrolimus
2	12/28/2022	4/6/2023	34	Female	GVHD	9/12/2023	23	Disease Progresssion; Deceased	Prednisone; Tacrolimus
3	7/13/2021	4/24/2023	53	Male	BOS	7/15/2025	55	Disease progression	Tacrolimus; MMF; Prednisone; Azithromycin; IVIG
4	1/2/2018	4/10/2023	51	Female	BOS	10/23/2025	154	Second Transplant	Tacrolimus; Prednisone; Azithromycin; MMF
5	7/28/2021	4/18/2023	73	Male	GVHD	5/30/2024	136	Improved	Ruxolitinib; Tacrolimus; Prednisone
6	1/4/2021	8/17/2023	66	Male	GVHD	8/27/2025	175	Disease progression; Deceased	Tacrolimus; Prednisone; Ruxolitinib; Budesonide
7	4/14/2022	8/23/2023	44	Female	BOS	Ongoing	45	Stable	Tacrolimus; MMF; Prednisone; Azithromycin
8	11/17/2022	8/24/2023	66	Female	BOS	1/12/2024	29	Disease Progression; Deceased	Tacrolimus; Azathioprine; Prednisone; Azithromycin
9	7/18/2019	7/20/2023	45	Male	BOS	7/21/2023	104	Second Transplant	Tacrolimus; MMF; Prednisone; Azithromycin
10	5/20/2021	7/20/2023	44	Female	BOS	7/21/2023	57	Off ECP; Stable	Tacrolimus; MMF; Prednisone
11	9/25/2018	8/15/2023	71	Male	CTCL	Ongoing	157	Stable	None
12	10/11/2023	3/21/2024	71	Female	GVHD	3/21/2024	29	Improved	Ruxolitinib; Prednisone

*Note:* Basic characteristics of the ECP patients included in this study. Samples utilized in this study were collected on the date indicated. The procedure number at which the sample was collected is also shown.

Abbreviations: BOS, Bronchiolitis Obliterans; GVHD, Graft Versus Host Disease; IVIG, Intravenous Immunoglobulin; MMF, Mycophenolate Mofetil.

While these results identified increased suppressive capacity in CD8 populations after ECP, it remained possible that the CD4+ CD25− responder cells were similarly affected by ECP. To this end, we have previously found that CD4+ T cell subsets respond differently to CD8 suppression, with some subsets showing pronounced resistance to suppression [31]. To determine if the increased suppression after ECP was due to differences in CD4+ T cells, CD8+ T cells, or both, we performed allo‐suppression assays. To do this, we isolated CD4+ CD25− responder cells from either healthy donors or ECP patients and attempted to suppress their proliferation with autologous or allogeneic cells. Using this approach, we found that CD8+ T cells from ECP patients were better able to suppress proliferation of allogeneic cells than autologous CD8+ T cells (Figure 3D). Conversely, CD8+ T cells from healthy controls exhibited almost no ability to suppress responder cells from ECP patients (Figure 3E). Taken together, these data suggest a complex relationship between CD8+ suppressors and CD4+ CD25− responders in patients undergoing ECP that warrants further investigation.

## 4 Discussion

To our knowledge this is the first study to explore the use of ECP in a murine model of EAE. Interestingly, there were brief reports from the early 2000s that suggested ECP could protect against EAE in a rat model [32, 33]. While these studies were more limited in their scope, they corroborate our findings and provide further argument for the use of ECP in autoimmune neuroinflammatory disease, so long as there is a significant T cell component to the disease. The observation that ECP successfully mitigated disease after symptom onset is important for potential future clinical applications as patients considering ECP will likely have advanced disease not controlled by other therapies. Importantly, small clinical trials in patients with MS have shown that although ECP cannot necessarily reverse longstanding damage associated with MS [34], it appeared to successfully prevent relapses [35]. Therefore, although the correlation between EAE and MS is not perfect, our data are in line with limited clinical observations. Of note, there is an ongoing clinical trial exploring ECP as a treatment modality in patients with RRMS that is currently enrolling patients (ClinicalTrials.gov ID NCT05168384).

It is important to note that delivery of ECP‐treated cells via intravenous injection led to reduced pathology in the CNS. The ability to exert a therapeutic effect in an isolated compartment while delivering the cells via simple injection is vital given the way in which ECP is performed clinically. Although our focus is on EAE, a model for MS, this study lays the groundwork for using ECP in other T cell‐mediated autoimmune neuroinflammatory disorders. This is particularly important as many such diseases are poorly understood, making targeted therapies difficult to pursue, leading to reliance on broadly immunosuppressive approaches. While effective, chronic immunosuppression can cause severe, potentially fatal side effects [36]. Thus, the true advantage of ECP is that it has the potential to be applied broadly and can be utilized without an in‐depth knowledge of disease pathophysiology. Our study fits into the work of others that look to expand the clinical use of this powerful, but underutilized, immunotherapy.

We found that the protective effect of ECP is highly specific to the antigen used to induce EAE. This is important as it suggests the effect of ECP is limited to the immunodominant response even when using different epitopes of the same protein (Figure 1C). This fits with the so‐called “dominant clone” hypothesis which suggests that ECP will induce immune tolerance to the predominant immunologic response; hence, it is not globally immunosuppressive. One of the aspects that makes treating MS so challenging is that the dominant epitope can change throughout the course of disease [37]. This makes exciting therapies such as chimeric autoantigen receptor T‐cells, used in other autoimmune disorders [38], difficult to apply. Thus, the advantage of ECP as a potential treatment for MS is that the therapy continues to work as the dominant antigen shifts.

To explore potential human correlations of the findings from our murine model, we explored functional changes in human CD8^+^ T‐cells in patients undergoing ECP. The observation that CD8^+^ T‐cells from ECP patients are more suppressive than those in healthy donors, and these suppressive effects are enhanced after initiating ECP, fit with the protective role of these cells seen in mice. Of course, it was possible that the increased suppression we observed was instead due to decreased “resistance” from the CD4^+^ T‐cells used in our assays. Interestingly, our allo‐suppression assays demonstrated that not only are CD8^+^ T‐cells from ECP patients more potent suppressors, but the CD4^+^ T‐cells from these same patients are more “resistant” to suppression (Figure 3D,E). There are many possible explanations for these findings, but it stands to reason that the effect of ECP is to enhance the suppressive function of CD8^+^ T‐cells to overcome the increased resistance of CD4^+^ T‐cells that may be secondary to many causes.

It is important to note the limitations of our human data. First, we included patients with diverse indications for ECP (Sezary syndrome, cellular rejection of a transplanted organ, graft vs. host disease) and varying therapy durations. Normal donors are not age/sex matched, and their medical history is only known insofar as their eligibility to donate blood. We also do not make comparisons to patients with similar conditions to those in the ECP group that are ECP naïve. Finally, we are not able to correlate qualitative and quantitative changes in CD8+ T‐cell populations to outcomes in patients with our current data set. To address these weaknesses, future work looking at phenotypic changes in T cell populations in individual patients over the course of ECP treatment (starting with a baseline, pre‐ECP sample) is ongoing in our laboratory.

## 5 Conclusions

The observation that ECP is dependent on the presence of CD8^+^ T‐cells provides mechanistic insight that has implications beyond EAE and MS. Of particular interest, CD8^+^ T‐cells with suppressive capacity may explain the therapeutic effect of ECP across a variety of diverse disease states and thus offer a unifying mechanism of action. Recent reports by others have implicated a unique population of suppressive CD8^+^ T‐cells in a contact hypersensitivity model of ECP [19]. Unlike CD4+ Tregs, which are easily identified by a common phenotype, suppressive CD8 cells are harder to define, making study comparisons difficult. Nevertheless, by understanding the cell population that mediates protection we can better identify disease states that may benefit from ECP. Future work exploring CD8^+^ suppressors is needed and will continue to shed light on these findings.

## Funding

This work was supported by the National Institutes of Health, 5UE5NS079173, P30CA086862; U.S. Department of Veterans Affairs, I01BX003677, I01CX002319.

## Conflicts of Interest

The authors declare no conflicts of interest.

## Supporting information


**Figure S1:** Representative flow cytometry plots for suppression assays. General gating strategy for all CD8+ suppression assays. Cells are gated on all events ➔ live cells ➔ singlets ➔ CD4+ T cells ➔ CFSE+ CD3+ cells. Representative plots for a non‐suppressed population (A) and suppressed population (B).

## Data Availability

The data that support the findings of this study are available from the corresponding author upon reasonable request.

## References

[1] D. J. Hanlon , C. L. Berger , and R. L. Edelson , “Photoactivated 8‐Methoxypsoralen Treatment Causes a Peptide‐Dependent Increase in Antigen Display by Transformed Lymphocytes,” International Journal of Cancer 78, no. 1 (1998): 70–75. 9724096 10.1002/(sici)1097-0215(19980925)78:1<70::aid-ijc12>3.0.co;2-9

[2] R. Edelson , C. Berger , F. Gasparro , et al., “Treatment of Cutaneous T‐Cell Lymphoma by Extracorporeal Photochemotherapy. Preliminary Results,” New England Journal of Medicine 316, no. 6 (1987): 297–303. 3543674 10.1056/NEJM198702053160603

[3] H. Hackstein , A. Kalina , B. Dorn , et al., “CD11c(+) Dendritic Cells Mediate Antigen‐Specific Suppression in Extracorporeal Photopheresis,” Clinical and Experimental Immunology 203, no. 2 (2021): 329–339. 33073358 10.1111/cei.13539 PMC7806418

[4] K. Tatsuno , T. Yamazaki , D. Hanlon , et al., “Extracorporeal Photochemotherapy Induces Bona Fide Immunogenic Cell Death,” Cell Death & Disease 10, no. 8 (2019): 578. 31371700 10.1038/s41419-019-1819-3 PMC6675789

[5] A. Ventura , A. Vassall , E. Robinson , et al., “Extracorporeal Photochemotherapy Drives Monocyte‐to‐Dendritic Cell Maturation to Induce Anticancer Immunity,” Cancer Research 78, no. 14 (2018): 4045–4058. 29764863 10.1158/0008-5472.CAN-18-0171

[6] T. S. Durazzo , R. E. Tigelaar , R. Filler , A. Hayday , M. Girardi , and R. L. Edelson , “Induction of Monocyte‐to‐Dendritic Cell Maturation by Extracorporeal Photochemotherapy: Initiation via Direct Platelet Signaling,” Transfusion and Apheresis Science 50, no. 3 (2014): 370–378. 24360371 10.1016/j.transci.2013.11.008 PMC4167725

[7] H. Jiang , S. I. Zhang , and B. Pernis , “Role of CD8+ T Cells in Murine Experimental Allergic Encephalomyelitis,” Science 256, no. 5060 (1992): 1213–1215. 1375398 10.1126/science.256.5060.1213

[8] J. Li , M. Zaslavsky , Y. Su , et al., “KIR(+)CD8(+) T Cells Suppress Pathogenic T Cells and Are Active in Autoimmune Diseases and COVID‐19,” Science 376, no. 6590 (2022): eabi9591. 35258337 10.1126/science.abi9591 PMC8995031

[9] M. P. Crawford , S. X. Yan , S. B. Ortega , et al., “High Prevalence of Autoreactive, Neuroantigen‐Specific CD8+ T Cells in Multiple Sclerosis Revealed by Novel Flow Cytometric Assay,” Blood 103, no. 11 (2004): 4222–4231. 14976054 10.1182/blood-2003-11-4025

[10] E. J. Baughman , J. P. Mendoza , S. B. Ortega , et al., “Neuroantigen‐Specific CD8+ Regulatory T‐Cell Function Is Deficient During Acute Exacerbation of Multiple Sclerosis,” Journal of Autoimmunity 36, no. 2 (2011): 115–124. 21257291 10.1016/j.jaut.2010.12.003 PMC3046327

[11] C. Vemulawada , P. S. Renavikar , M. P. Crawford , S. Steward‐Tharp , and N. J. Karandikar , “Disruption of IFNgamma, GZMB, PRF1, or LYST Results in Reduced Suppressive Function in Human CD8+ T Cells,” Journal of Immunology (Baltimore, Md: 1950) 212, no. 11 (2024): 1722–1732. 38607279 10.4049/jimmunol.2300388 PMC11105984

[12] P. S. Renavikar , S. Sinha , A. A. Brate , et al., “IL‐12‐Induced Immune Suppressive Deficit During CD8+ T‐Cell Differentiation,” Frontiers in Immunology 11 (2020): 568630. 33193343 10.3389/fimmu.2020.568630 PMC7657266

[13] K. Cunnusamy , E. J. Baughman , J. Franco , et al., “Disease Exacerbation of Multiple Sclerosis Is Characterized by Loss of Terminally Differentiated Autoregulatory CD8+ T Cells,” Clinical Immunology 152, no. 1–2 (2014): 115–126. 24657764 10.1016/j.clim.2014.03.005 PMC4024444

[14] N. R. York , J. P. Mendoza , S. B. Ortega , et al., “Immune Regulatory CNS‐Reactive CD8+T Cells in Experimental Autoimmune Encephalomyelitis,” Journal of Autoimmunity 35, no. 1 (2010): 33–44. 20172692 10.1016/j.jaut.2010.01.003 PMC2878858

[15] S. B. Ortega , V. P. Kashi , A. F. Tyler , K. Cunnusamy , J. P. Mendoza , and N. J. Karandikar , “The Disease‐Ameliorating Function of Autoregulatory CD8 T Cells Is Mediated by Targeting of Encephalitogenic CD4 T Cells in Experimental Autoimmune Encephalomyelitis,” Journal of Immunology (Baltimore, Md: 1950) 191, no. 1 (2013): 117–126. 23733879 10.4049/jimmunol.1300452 PMC3691355

[16] A. W. Boyden , A. A. Brate , L. M. Stephens , and N. J. Karandikar , “Immune Autoregulatory CD8 T Cells Require IFN‐Gamma Responsiveness to Optimally Suppress Central Nervous System Autoimmunity,” Journal of Immunology (Baltimore, Md: 1950) 205, no. 2 (2020): 359–368. 32532836 10.4049/jimmunol.2000211 PMC7343581

[17] A. A. Brate , A. W. Boyden , F. R. Itani , L. L. Pewe , J. T. Harty , and N. J. Karandikar , “Therapeutic Intervention in Relapsing Autoimmune Demyelinating Disease Through Induction of Myelin‐Specific Regulatory CD8 T Cell Responses,” Journal of Translational Autoimmunity 2 (2019): 100010. 32161909 10.1016/j.jtauto.2019.100010 PMC7065686

[18] A. W. Boyden , A. A. Brate , and N. J. Karandikar , “Early IFNgamma‐Mediated and Late Perforin‐Mediated Suppression of Pathogenic CD4 T Cell Responses Are Both Required for Inhibition of Demyelinating Disease by CNS‐Specific Autoregulatory CD8 T Cells,” Frontiers in Immunology 9 (2018): 2336. 30356717 10.3389/fimmu.2018.02336 PMC6189364

[19] O. Hequet , A. Nosbaum , A. Guironnet‐Paquet , et al., “CD8(+) T Cells Mediate Ultraviolet A‐Induced Immunomodulation in a Model of Extracorporeal Photochemotherapy,” European Journal of Immunology 50, no. 5 (2020): 725–735. 32012249 10.1002/eji.201948318

[20] H. Budde , S. Kolb , L. Salinas Tejedor , et al., “Modified Extracorporeal Photopheresis With Cells From a Healthy Donor for Acute Graft‐Versus‐Host Disease in a Mouse Model,” PLoS One 9, no. 8 (2014): e105896. 25148404 10.1371/journal.pone.0105896 PMC4141828

[21] C. S. Constantinescu , N. Farooqi , K. O'Brien , and B. Gran , “Experimental Autoimmune Encephalomyelitis (EAE) as a Model for Multiple Sclerosis (MS),” British Journal of Pharmacology 164, no. 4 (2011): 1079–1106. 21371012 10.1111/j.1476-5381.2011.01302.x PMC3229753

[22] R. M. Ransohoff , “Animal Models of Multiple Sclerosis: The Good, the Bad and the Bottom Line,” Nature Neuroscience 15, no. 8 (2012): 1074–1077. 22837037 10.1038/nn.3168 PMC7097342

[23] A. W. Boyden , A. A. Brate , and N. J. Karandikar , “Novel B Cell‐Dependent Multiple Sclerosis Model Using Extracellular Domains of Myelin Proteolipid Protein,” Scientific Reports 10, no. 1 (2020): 5011. 32193439 10.1038/s41598-020-61928-w PMC7081236

[24] A. A. Brate , A. W. Boyden , I. J. Jensen , V. P. Badovinac , and N. J. Karandikar , “A Functionally Distinct CXCR3(+)/IFN‐Gamma(+)/IL‐10(+) Subset Defines Disease‐Suppressive Myelin‐Specific CD8 T Cells,” Journal of Immunology (Baltimore, Md: 1950) 206, no. 6 (2021): 1151–1160. 33558376 10.4049/jimmunol.2001143 PMC8059448

[25] C. R. Wilhelm , M. A. Upadhye , K. L. Eschbacher , N. J. Karandikar , and A. W. Boyden , “Proteolipid Protein‐Induced Mouse Model of Multiple Sclerosis Requires B Cell‐Mediated Antigen Presentation,” Journal of Immunology (Baltimore, Md: 1950) 211, no. 6 (2023): 944–953. 37548478 10.4049/jimmunol.2200721 PMC10528642

[26] V. P. Kashi , S. B. Ortega , and N. J. Karandikar , “Neuroantigen‐Specific Autoregulatory CD8+ T Cells Inhibit Autoimmune Demyelination Through Modulation of Dendritic Cell Function,” PLoS One 9, no. 8 (2014): e105763. 25144738 10.1371/journal.pone.0105763 PMC4140828

[27] E. Gatza , C. E. Rogers , S. G. Clouthier , et al., “Extracorporeal Photopheresis Reverses Experimental Graft‐Versus‐Host Disease Through Regulatory T Cells,” Blood 112, no. 4 (2008): 1515–1521. 18411417 10.1182/blood-2007-11-125542 PMC2515148

[28] S. D. Miller and W. J. Karpus , “Experimental Autoimmune Encephalomyelitis in the Mouse,” Current Protocols in Immunology Chapter 15 (2007): 15.1.1–15.1.18. 10.1002/0471142735.im1501s77 PMC2915550 18432984

[29] A. Maeda , A. Schwarz , K. Kernebeck , et al., “Intravenous Infusion of Syngeneic Apoptotic Cells by Photopheresis Induces Antigen‐Specific Regulatory T Cells,” Journal of Immunology (Baltimore, Md: 1950) 174, no. 10 (2005): 5968–5976. 15879089 10.4049/jimmunol.174.10.5968

[30] A. Maeda , A. Schwarz , A. Bullinger , A. Morita , D. Peritt , and T. Schwarz , “Experimental Extracorporeal Photopheresis Inhibits the Sensitization and Effector Phases of Contact Hypersensitivity via Two Mechanisms: Generation of IL‐10 and Induction of Regulatory T Cells,” Journal of Immunology (Baltimore, Md: 1950) 181, no. 9 (2008): 5956–5962. 18941184 10.4049/jimmunol.181.9.5956

[31] M. P. Crawford , S. Sinha , P. S. Renavikar , N. Borcherding , and N. J. Karandikar , “CD4 T Cell‐Intrinsic Role for the T Helper 17 Signature Cytokine IL‐17: Effector Resistance to Immune Suppression,” Proceedings of the National Academy of Sciences of the United States of America 117, no. 32 (2020): 19408–19414. 32719138 10.1073/pnas.2005010117 PMC7430972

[32] G. Cavaletti , P. Perseghin , M. Dassi , et al., “Extracorporeal Photochemotherapy Reduces the Severity of Lewis Rat Experimental Allergic Encephalomyelitis Through a Modulation of the Function of Peripheral Blood Mononuclear Cells,” Journal of Biological Regulators and Homeostatic Agents 18, no. 1 (2004): 9–17. 15323355

[33] G. Cavaletti , P. Perseghin , F. Buscemi , et al., “Immunomodulating Effects of Extracorporeal Photochemotherapy in Rat Experimental Allergic Encephalomyelitis,” International Journal of Tissue Reactions 23, no. 1 (2001): 21–31. 11392060

[34] A. M. Rostami , R. A. Sater , S. J. Bird , et al., “A Double‐Blind, Placebo‐Controlled Trial of Extracorporeal Photopheresis in Chronic Progressive Multiple Sclerosis,” Multiple Sclerosis 5, no. 3 (1999): 198–203. 10408721 10.1177/135245859900500310

[35] G. Cavaletti , P. Perseghin , M. Dassi , et al., “Extracorporeal Photochemotherapy: A Safety and Tolerability Pilot Study With Preliminary Efficacy Results in Refractory Relapsing‐Remitting Multiple Sclerosis,” Neurological Sciences 27, no. 1 (2006): 24–32. 16688596 10.1007/s10072-006-0561-7

[36] L. Klotz , J. Havla , N. Schwab , et al., “Risks and Risk Management in Modern Multiple Sclerosis Immunotherapeutic Treatment,” Therapeutic Advances in Neurological Disorders 12 (2019): 1756286419836571. 30967901 10.1177/1756286419836571 PMC6444778

[37] V. K. Tuohy and R. P. Kinkel , “Epitope Spreading: A Mechanism for Progression of Autoimmune Disease,” Archivum Immunologiae et Therapiae Experimentalis (Warsz) 48, no. 5 (2000): 347–351. 11140461

[38] S. M. Reincke , N. von Wardenburg , M. A. Homeyer , et al., “Chimeric Autoantibody Receptor T Cells Deplete NMDA Receptor‐Specific B Cells,” Cell 186, no. 23 (2023): 5084–5097. 37918394 10.1016/j.cell.2023.10.001

