# Supplementary material for: Suppressive CD8+ T‐Cells Are Key Cellular Mediators of Extracorporeal Photopheresis
Source: J Clin Apher. 2026 Feb 7;41(1):e70094. doi: 10.1002/jca.70094 (PMC12882035; doi:10.1002/jca.70094)
Supplement: Supplementary file 1 — Figure S1: Representative flow cytometry plots for suppression assays. General gating strategy for all CD8+ suppression assays. Cells are gated on all events ➔ live cells ➔ singlets ➔ CD4+ T cells ➔ CFSE+ CD3+ cells. Representative plots for a non‐suppressed population (A) and suppressed population (B). [file JCA-41-e70094-s001.pptx]

## Slide 1
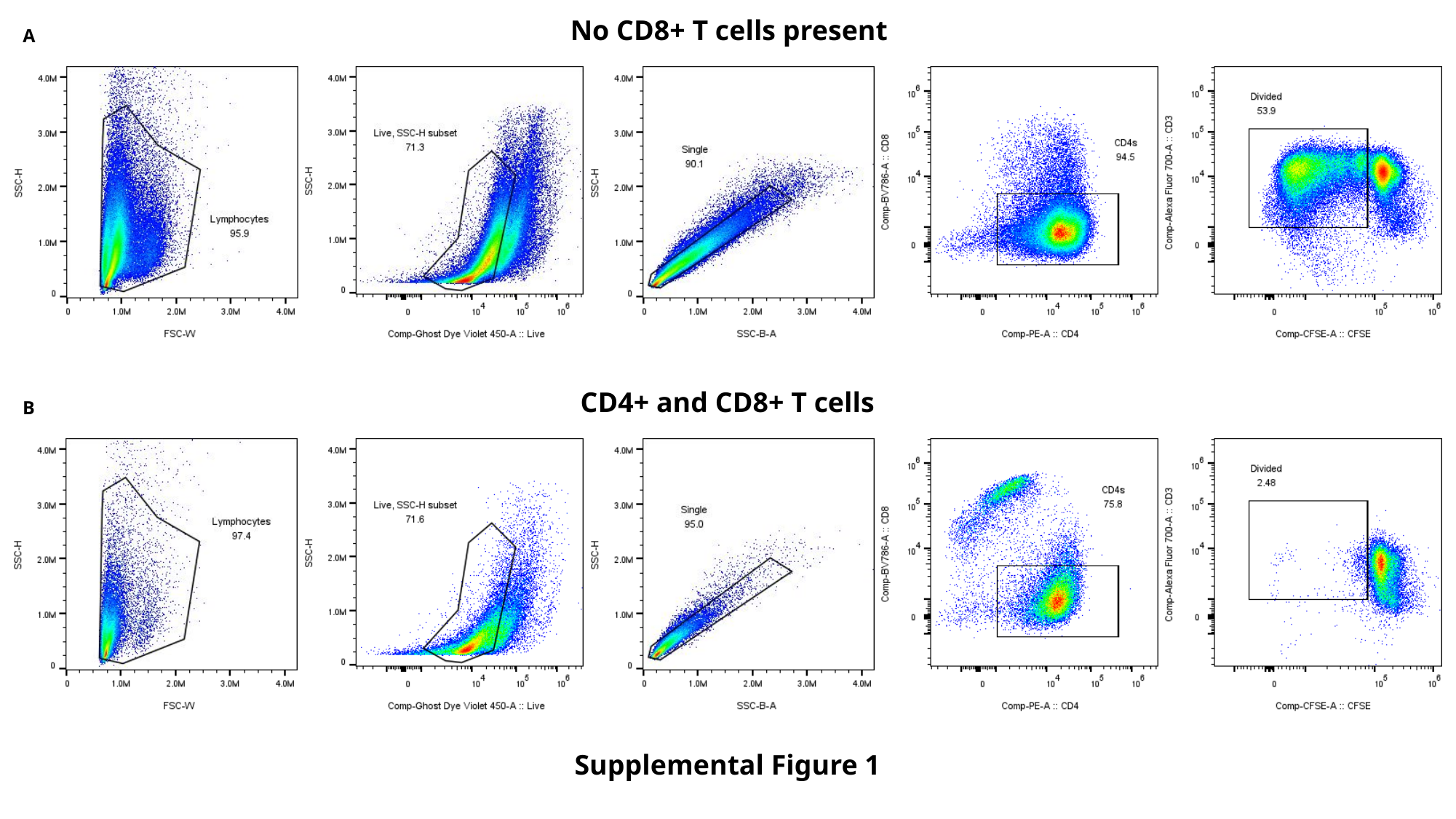

No CD8+ T cells present
A
CD4+ and CD8+ T cells
B
Supplemental Figure 1

## Slide 2
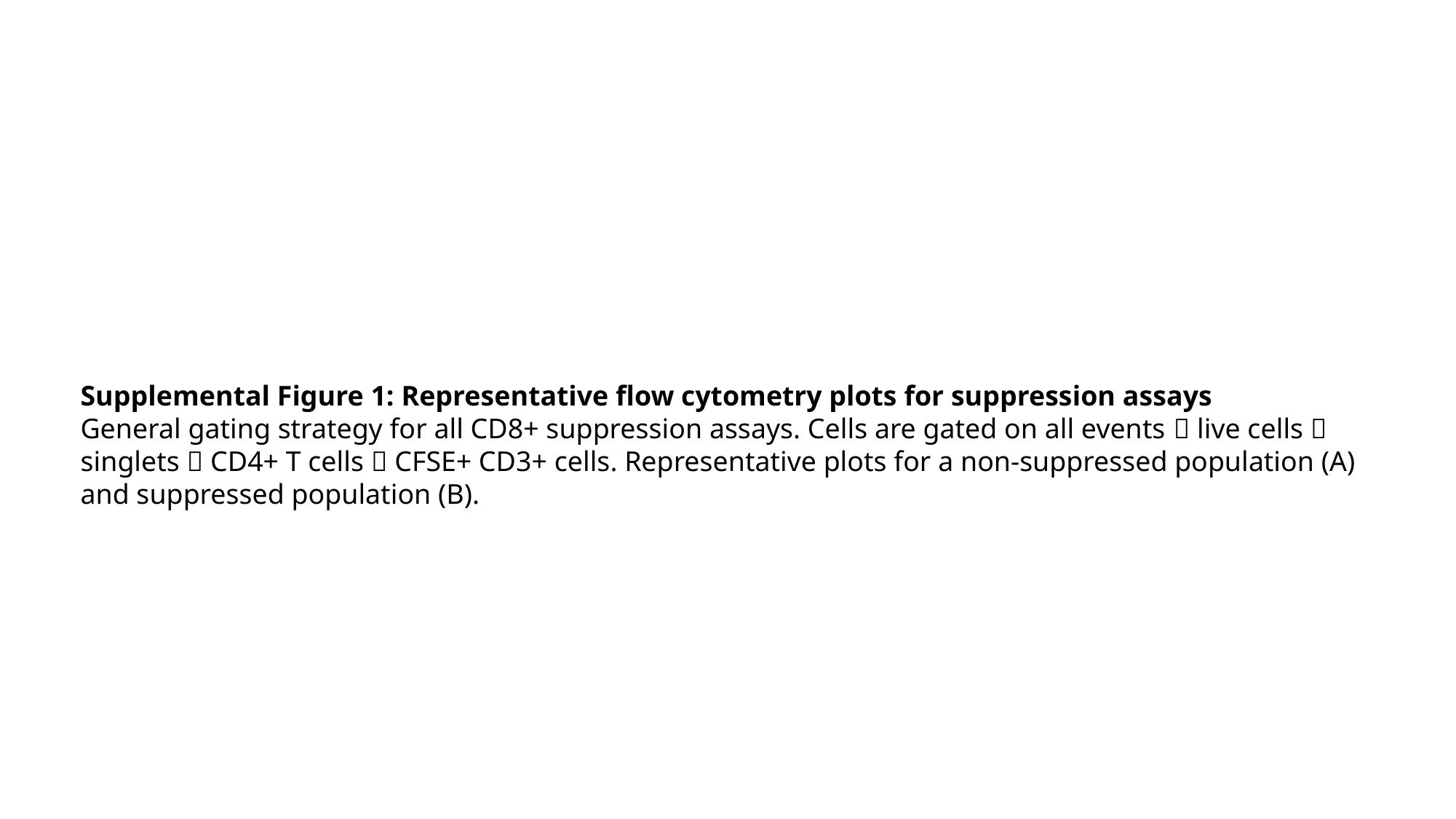

Supplemental Figure 1: Representative flow cytometry plots for suppression assays
General gating strategy for all CD8+ suppression assays. Cells are gated on all events  live cells  singlets  CD4+ T cells  CFSE+ CD3+ cells. Representative plots for a non-suppressed population (A) and suppressed population (B).
